# Attention-sensitive signalling by 7- to 20-month-old infants in a comparative perspective

**DOI:** 10.3389/fpsyg.2024.1257324

**Published:** 2024-03-18

**Authors:** Mawa Dafreville, Michèle Guidetti, Marie Bourjade

**Affiliations:** ^1^CLLE, Université de Toulouse, CNRS, Toulouse, France; ^2^Institut Universitaire de France, Paris, France

**Keywords:** sensory modality, infant-directed speech, visual attention, mother-infant dyads, language acquisition, language evolution

## Abstract

*Attention-sensitive signalling* is the pragmatic skill of signallers who adjust the modality of their communicative signals to their recipient’s attention state. This study provides the first comprehensive evidence for its onset and development in 7-to 20-month-olds human infants, and underlines its significance for language acquisition and evolutionary history. Mother-infant dyads (*N* = 30) were studied in naturalistic settings, sampled according to three developmental periods (in months); [7–10], [11–14], and [15–20]. Infant’s signals were classified by dominant perceptible sensory modality and proportions compared according to their mother’s *visual attention*, *infant-directed speech* and *tactile contact*. Maternal visual attention and infant-directed speech were influential on the onset and steepness of infants’ communicative adjustments. The ability to inhibit silent-visual signals towards visually inattentive mothers (*unimodal* adjustment) predated the ability to deploy audible-or-contact signals in this case (*cross-modal* adjustment). Maternal scaffolding of infant’s early pragmatic skills through her infant-directed speech operates on the facilitation of infant’s *unimodal* adjustment, the preference for oral over gestural signals, and the audio-visual combinations of signals. Additionally, breakdowns in maternal visual attention are associated with increased use of the audible-oral modality/channel. The evolutionary role of the sharing of attentional resources between parents and infants into the emergence of modern language is discussed.

## Introduction

Besides sign languages that rely on silent-visual signs, all other natural languages are both spoken and gestured, i.e., they are multimodal at both the production (gestural, facial or oral) and the perception (audible, visual or tactile) levels (Wilcox and Xavier, 2013; Vigliocco et al., 2014). Despite such a universality, science has reached no consensus so far about the evolutionary history of voluntary control over the vocal cords in early humans, apart from all other primates (Locke, 1996; Bergman et al., 2019). In line with this discontinuity in evolution, early pragmatics in human infants developing language has shown consistent evidence for gestures predating vocalisations in terms of intentional and conventional uses (Bates et al., 1975; Guidetti et al., 2014; Zlatev and McCune, 2014; Donnellan et al., 2019). Although vocal behaviour has the potential to attain the recipient even in the case of visual inattention, experiencing visual breakdowns might be necessary for signallers to switch from gestures to vocalisations, both in language acquisition (Oller et al., 2016; Bourjade et al., 2023) and in the course of evolution (Falk, 2004; Locke, 2006; Mehr and Krasnow, 2017). However, no study has provided comprehensive evidence for the development of such an *attention-sensitive signalling* in human infants, although experiments have pointed out consistent links between vocalizations and the *attention* provided by the adult recipient (Liszkowski et al., 2008; Igualada et al., 2015; Wu and Gros-Louis, 2017; Bourjade et al., 2023). The present work articulates the framework of evolutionary developmental psychology (Bjorklund and Pellegrini, 2000; Frankenhuis and Tiokhin, 2018) with evidence-based research in early pragmatics to address *attention-sensitive signalling* in 7- to 20-month-old infants observed in naturalistic contexts.

Evolutionary developmental psychology encompasses the comparative study of developmental stages in multiple related species (Bjorklund and Pellegrini, 2000; Locke and Bogin, 2006). Specific hypotheses stipulate that attentional resources shared between parents and infants in early *hominina* belong to the selective forces involved in the emergence of proto-linguistic vocal substrates (Locke, 1996, 2006; Falk, 2004; Mehr and Krasnow, 2017). It is thought that after the bipedalism breakthrough, increased physical distance between mothers and infants prompted mothers to vocalise (Falk, 2004) or sing (Mehr and Krasnow, 2017) to soothe their dependent offspring in the absence of physical contact. On the infant’s side, vocal behaviour is likely to attract attention, and natural selection may have favoured infants producing sophisticated and tactical vocalizations (Locke, 1996, 2006). Canonical babbling, which is the first emergence of syllables in 7-month-old infants, may progressively have constituted an indicator of the infant’s good health and may have encouraged more parental investment (Locke, 1996, 2006; Locke and Bogin, 2006; Oller et al., 2016). According to these theoretical accounts, the prominence of audible/vocal communication in the human lineage may have emerged within caregiver-infant communication and attentional exchanges.

Non-human primates also communicate using distinct sensory modalities. Communication repertoires consist of signals classified by the dominant sensory modality of *perception*, stemming from *audible*, *silent-visual* and *contact* signals; in the great apes (Pika et al., 2005; Hobaiter and Byrne, 2011; Byrne et al., 2017) and in monkeys (Gupta and Sinha, 2019; Fröhlich and van Schaik, 2020; Molesti et al., 2020). Most of these signals show a visual component, but when they additionally make sound or involve physical contact with the recipient, they are, respectively, considered as *audible* and *contact* signals. Lab-based experiments as well as field observations indicate that at least chimpanzees and baboons can adjust the perceptible sensory modality of their signals to their recipient’s attention state (Leavens et al., 2010; Hobaiter and Byrne, 2011; Bourjade et al., 2014; Molesti et al., 2017). In addition, the sensory channel conveying information is also relevant for studying communication and language in an evolutionary perspective (Fröhlich and van Schaik, 2020). Non-voiced oral signals have for example been considered as potential precursors of human speech (e.g.: *atypical sounds*, Meguerditchian et al., 2014; Bergman et al., 2019). Yet, there is a dearth of comparative research focusing on these non-voiced oral signals in human infants (e.g.: *raspeberries*, Oller, 2000). The present study uses an ethological coding that will fill an important gap in knowledge and complement the few human studies that have recently undertaken comparisons with non-human primate gestural communication (Kersken et al., 2019; Rodrigues et al., 2021; Bourjade et al., 2023).

Preverbal infants develop a differential use of sensory modalities to communicate with surrounding adults. Around 6 months, infants use *body movements* to delineate early forms of interaction with an adult (Scola et al., 2015; Bourjade et al., 2023). *Smiles* and *coos* coordinated with gaze is standard communication before 8 months of age, and can be sensitive to the adult’s attention and interaction (Jones et al., 1991; Jones and Hong, 2001; Crown et al., 2002; Fröhlich and van Schaik, 2020; Northrup and Iverson, 2020; Bourjade et al., 2023). A great deal of research has investigated the communicative function of shared reference by considering *deictic gestures* (i.e., *giving*, *showing, reaching*, *index-finger pointing* gestures) that emerge progressively during the second half of the first year (Liszkowski et al., 2006; Rodríguez et al., 2015; Murillo et al., 2021). Infants also vocalise from birth, although non-speech vocalisations dominate the repertoire until 8 months of age (Oller, 2000). Repertoires include proper vocalisations that activate the vocal cords (e.g., *babbling and vowel-like sounds)* but also some non-voiced sounds made without activation of the vocal cords (e.g.: *raspeberries*, Oller, 2000). Experimental research has also evidenced that infants couple vocalizations to pointing gestures as a function of the *attention* provided by the adult recipient (Liszkowski et al., 2008; Igualada et al., 2015; Wu and Gros-Louis, 2017) and that the coupling is associated with subsequent language outcomes (Wu and Gros-Louis, 2014, 2017; Igualada et al., 2015).

Most of the above experimental research has focused on declarative pointing tasks in which the infant is invited to show different objects to an adult who varies her visual attention and responsiveness. Liszkowski et al. (2008) initially showed that 12-month-old infants decreased the frequency of pointing gestures towards visually inattentive adults as compared to visually attentive ones. By comparing infants of 12 and 18 months of age, they found that only 18-month-old infants produced more vocalisations when the adults were not visually attentive and not responsive than when they were visually attentive and responsive (Liszkowski et al., 2008). Igualada et al. (2015) found that 12-month-old infants used more pointing–vocal coupling when the adults showed visual attention than when they did not while some non-experimental studies found the opposite pattern, with more pointing–vocal coupling when the adults did not show visual attention than when they did in 10-to 13-month-old infants (Gros-Louis and Wu, 2012; Wu and Gros-Louis, 2014). Despite these mixed results, all these studies pointed out the link between vocal communication and parental attention, but none has considered broad repertoires of communication in naturalistic contexts.

Two recent comparative studies on *attention-sensitive signalling* (i.e., the capacity of signallers to flexibly adjust the sensory modality of their communicative signals to the recipient’s attention state) have focused on broader repertoires of gestures but did not take vocalizations into account. Rodrigues et al. (2021) studied the adjustment of audible, silent-visual and contact gestures by 7- to 12-month-old infants to their recipient’s visual attention in nurseries and found contact gestures to be preferred in the event of visual inattention. Using a similar coding, Dafreville et al. (2021) used two descriptors of attention-sensitive signalling to study developmental trajectories in wild chimpanzees: *unimodal* and *cross-modal adjustments*. They found that immature chimpanzees preferentially used silent-visual gestures in front of a visually attentive mother, i.e., *unimodal adjustment*, and switched communication modalities when her visual attention was unavailable; juveniles preferred using contact gestures and adolescents preferred using audible gestures, i.e., *cross-modal adjustment* (Dafreville et al., 2021). While *unimodal adjustment* describes the capacity to avoid communication mismatch, *cross-modal adjustment* stands as a proxy for the capacity to switch modalities in order to favour communication match.

The present study explores for the first time these capacities in human infants within the comparative framework described above. The study was conducted in the naturalistic context of the home and was therefore not experimental. Instead, we conducted ecological observations, followed by an ethological coding anchored in a multimodal approach to communication (Vigliocco et al., 2014; Byrne et al., 2017; Kersken et al., 2019). Our aim was to quantify the infants’ attention-sensitive signalling across spontaneous variations of maternal *visual attention* (categorised as visually attentive/inattentive) from video footages filmed in the home. We first explored the different maternal and contextual variables likely to affect the infant’s production of signals (the results of this exploratory analyses are presented as supplementary information). We then tested specific predictions derived from the comparative framework of attention-sensitive signalling. Namely, we tested infants’ capability for *unimodal* and *cross-modal* adjustments by investigating the use of signals between the conditions where the mother’s visual attention was available or unavailable. If infants show *unimodal* adjustment, they will show fewer silent-visual signals when the mother is not visually attentive than when she is attentive. If infants show *cross-modal* adjustment, they will produce more audible-or-contact than visual signals when the mother is visually inattentive compared to when she is attentive. Following Falk (2004), we also expected the mother’s infant-directed speech (IDS) to provide a compensatory form of attention to the infant, hence decreasing the infant’s signalling rates in *cross-modal* adjustment. If both predictions are true, we should observe infants producing more audible-or-contact signals than visual signals when the mother is visually inattentive, but in smaller proportions when the mother is talking to her infant (IDS) than not talking. Therefore, and in congruence with exploratory analyses that led to identify IDS as an influential factor, we conducted data analysis on two separate data sets; (i) with silent mothers, and (ii) with talking mothers.

Finally, we documented the *ways* infants were producing cross-modal adjustment with two additional analyses that targeted the evo-devo account of language emergence. We compared the use of tactile and audible gestures and oral signals when the mother was not visually attentive to establish infants’ preference for oral over gestural signals. Additionally, we explored if infants were using multimodal combinations in a strategic way, namely for *cross-modal* adjustment. Infants were aged 7 to 20 months in order to document developmental effects, and each analysis was conducted on the following age categories (in months): [7–10], [11–14], and [15–20].

## Methods

### Participants

The sample included 30 mother-infant dyads (11 girls, 19 boys; mean age: 12.93 months; range: 7 to 20.3 months of age). Mother-infant dyads came mainly from urban areas. A pre-home visit with the purpose of mutual familiarisation allowed the observer to briefly inform the mother about the study and to ask her a few questions about her infant (family configuration, type of childcare, etc.) using a short demographic questionnaire. Mostly, they were families with first-born infants (*n* = 18 out of 30 mother-infant dyads, 60%) and the average number of siblings per infant observed was 1.87. The mother’s age varied between 27 and 40 years. The mother had a high level of formal education, as they all had graduate degrees (at least bachelor level). Most of the infants were totally breastfed from birth to 6 months of age (*n* = 24 out of 30 mother-infant dyads, 80%). Nine individuals also experienced health difficulties at their birth (i.e., preterm; difficult childbirth; asthma).

In this cross-sectional study, mother-infant dyads were *a priori* allocated to three age groups: 7 to 10 months of age, 11 to 14 months of age and 15 to 20 months of age (see Table 1 for further details). The rationale was to set a first category that would encompass the onset of distal pointing gestures, which can be delayed by several weeks in some infants (Mccune and Zlatev, 2015). Then, we set up a middle age category with all infants able to point and starting to combine gestures and vocalizations (Donnellan et al., 2019). The third category comprised older infants able to combine vocalizations with pointing in strategic ways (Liszkowski et al., 2008).

**Table 1:** Number of human infants included in the study (mean age ± SD) by sex and age category.

Age category (in months)	[7–10]	[11–14]	[15–20]	Total
Females	**6** (8.83 ± 1.03)	**3** (12.78 ± 0.99)	**2** (18.53 ± 1.59)	**11** (11.67 ± 3.95)
Males	**5** (8.80 ± 1.09)	**9** (13.11 ± 0.91)	**5** (16.41 ± 0.93)	**19** (12.84 ± 2.99)
**Total**	**11** (8.81 ± 1.00)	**12** (13.03 ± 0.90)	**7** (17.01 ± 1.44)	**30** (12.41 ± 3.36)

### Procedure and design

Dyads were observed in naturalistic conditions at home during the context of meals (lunch, dinner, afternoon snack time). As visits aimed at accessing the most natural conditions, the observations took place whether other members of the family were present or not. During the observations, the observer stayed as much as possible on the side-lines and was not engaged in any interaction with the mother or the infant during the observation. Each dyad was observed twice for on average 1 h, 1 month apart. The recording started on average 15 min before the start of the meal (taking care to ensure that the infant was awake). Both visits were made so that the infant remained in the same age group during the two observations. The visits were filmed using two cameras: a camera (PANASONIC HC-V770) mounted on a tripod, providing an overview of the room and the movements of the mother in particular; a second camera (HANDYCAM HDR-CX625) hand-held by the observer, who focused only on the infant and followed him/her. All the mother-infant dyads were kept for further analysis, even if there was only one observation per infant (three infants were not observed twice).

### Ethical procedure

During the first visit, the parents of infant participants completed a consent form on behalf of their infants and were questioned about their profession, the sleep rhythm and natural eating habits of their infant. During the second visit, the experimenter added an amendment to the initial signed consent in which the mother authorised the researchers to study her own behaviour in addition to that of her infant. The video and coding data were anonymised and stocked on external drives stored in safe places locked until 15 years after the end of the study, at which time they will be destroyed.

### Data collection

Quantitative behavioural data were collected on videos using an individual focal sampling approach on the infant (Altmann, 1974). One infant was observed at a time and the infant’s communicative signals were recorded continuously over 3 focuses of 5 min where each focus was randomly selected before, during and after feeding per visit (1 and 2). When it was impossible to have one focus before or after the meal because the observation did not cover these three times, we replaced the missing meal time (for example ‘after meal’) by a second one from the same meal time (‘before meal’ in our example).

### Behavioural sampling and coding procedure

The infant’s communicative signals were coded in ELAN software (Version 5.2) following the repertoire in Supplementary Table S1. The repertoire included about 23 behaviours classified into three categories based on their dominant sensory modality of perception, i.e., visual, audible and tactile. In the repertoire, we considered that *audible signals* encompassed oral and gestural signals, while *silent-visual* and *tactile* signals were exclusively gestural (see Table 2; Supplementary Table S1 for further details).

**Table 2:** Brief categorisation of the communicative signals according to the signal modality and channel of production.

Perception modality	Production channel	Sub-groups of communicative signals
Silent-visual	Gestural	Body movements (circular movement of the limbs; synchronised trunk/members movement; swagger) Deictic gestures (whole-hand pointing gesture; index-pointing; reaching attempt; giving; receiving; showing) Conventional gestures Facial expressions (smiling)
Tactile	Gestural	Gestures involving the infant touching his/her mother with at least one body part (except with the feet)
Audible	Oral	Non-voiced sounds (mouth sounds and laughs ^(1)^) Voiced sounds (vowel-like sounds; grunts; babbling; cries; pseudo-words; words; succession of words)
	Gestural	Audible gestures (sound made by manual or bodily actions with or without an object)

The detailed repertoire of behaviours is available in the Supplementary Table S1. (1) laughs were included in non-voiced sounds because they were defined as being smiles with vocal or non-vocal sounds lasting more than 1 s, interspersed by silences during which the infant breathes in and so they are not necessarily produced with the vocal cords.

For each signal, we recorded the following information: (1) the sensory modality of perception (audible, tactile or silent-visual), (2) the production channel (gestural or oral), (3) if the signal was combined with another signal of another sensory modality, (4) the presence of another recipient closer than the mother (i.e., non-maternal recipient), (5) the attention state of the mother and of the non-maternal recipient if applicable. To define the recipient attention state, we recorded the attentional variables of interest below (for more details, see Supplementary Table S2): *(1) Visual attention* was recorded when the mother had her head directed at her infant from narrow to large angles, the geometric lines of sight of the two individuals being able to cross each other; *(2) Infant Directed Speech* was recorded when the mother was talking to her infant; *(3) Physical contact* was recorded when the mother had at least one body part in physical contact with her infant. *Auditory attention*, i.e., the possibility that the mother can hear her infant, was considered always present as we could not determine its absence. The *distance to the mother* was also coded and referred to the distance between the mother and the infant; *close*: the mother stands closer than an arm’s length from her infant; *far*: the mother stands farther than an arm’s length from her infant; *out-of-sight*: the mother is totally out of sight of her infant. Other potential confound variables were coded and detailed in the Supplementary material.

We also accounted for the various multimodal combinations defined as the combination of signals of different sensory modalities. Among these multimodal combinations, we distinguished between *distal combinations* (combination of audible and silent-visual signals without contact gestures) and *proximal combinations* (combination of at least a contact gesture and at least one other signal modality). A combination was recorded at each overlap between one signal and another one except if the time between the end of the first signal (over time) and the beginning of the second one was longer than 200 milliseconds. Because we focused on maternal attentional state in this study, all the infant signals potentially directed to a non-maternal recipient were removed.

### Inter-rater reliability

The videos were entirely coded by one main coder, who was therefore used as the reference for training a second blind coder. The second coder trained for 1 month, then blindly coded 20% of the dataset. Reliability was assessed through Cohen’s Kappa statistics. The second coder reached a Cohen’s Kappa coefficient of almost 0.80 with the first coder for the infants’ visual communicative behaviours; Cohen’s Kappa ranged from 0.72 to 0.83 across the infants’ audible signals (Supplementary Table S3). Agreement for maternal attention was high (Cohen’s k > 0.80; Supplementary Table S4).

### Statistical analysis

The first part of statistical analyses was exploratory. Contextual factors from the observations, mixing maternal attention states and possible confound variables were added in Generalised Linear Mixed Models on proportion data (Bolker et al., 2009; Harrison, 2015). The goal was to identify the confound variables to control for as well as the main fixed effects of maternal attention state. The process of model fitting, together with the output is presented as Supplementary material. Controlling for confound variables, we found that maternal visual attention, maternal infant-directed speech (IDS) and infant’s age were the three independent variables that explained most of the variance observed in the infants’ use of signal modality.

The second part of the statistical analyses aimed at testing predictions of *unimodal* and *cross-modal* adjustment. Because the sample size was small (30 mother-infant dyads) and the dependent variables did not follow normal distributions, we used non-parametric statistics to test defined hypotheses (Siegal and Castellan, 1988), including Bonferroni corrections in cases of multiple comparisons. All the non-parametric tests used to compare infant signalling depending on the mother’s visual attention were conducted separately by mother’s *infant-directed speech* (IDS, yes/no) and infant’s age (7–10; 11–14; 15–20). Permutation tests were used in each age group to test if the infants produced significantly (i) fewer silent-visual gestures when the mothers did not show visual attention than when they did, and (ii) more audible-or-contact-gestures when the mothers did not show visual attention than when they did (Dafreville et al., 2021). Fischer’s exact tests were used to test the dependency between maternal visual attention and infant signal modality. We expected infants to produce significantly more audible-or-contact signals than silent-visual gestures when the mothers did not show visual attention than when they did, in each age range (Dafreville et al., 2021).

In the third part of the analyses, we used permutation tests and Fisher’s exact tests to examine (i) the preferential use of oral signals against other signals when the mothers did not show visual attention and, (ii) the use of multimodal combinations according to maternal visual attention. All the tests were conducted using R v3.6.1 software^1^ with *p*-values equal to or lower than 0.05 required for significance. All the statistical tests were two-tailed except when specified.

## Results

Overall, we coded a total of 9,245 communicative signals. After removing all the signals potentially oriented towards non-maternal recipients, we were able to reliably analyse 8,367 signals: 192 tactile signals, 1,422 visual signals and 6,753 audible signals of which 4,658 were oral signals.

Rather than physical contact, the maternal visual attention, her infant-directed speech and the infant’s age were the most likely determining factors of signal production (for details, see Supplementary Table S6). That is why, all the non-parametric tests detailed below were conducted twice, on data (i) with maternal IDS (‘talking mother’), and (ii) without it (‘silent mother’).

### Unimodal adjustment: does the use of silent-visual gestures vary according to maternal visual attention and IDS?

**Figure 1:**
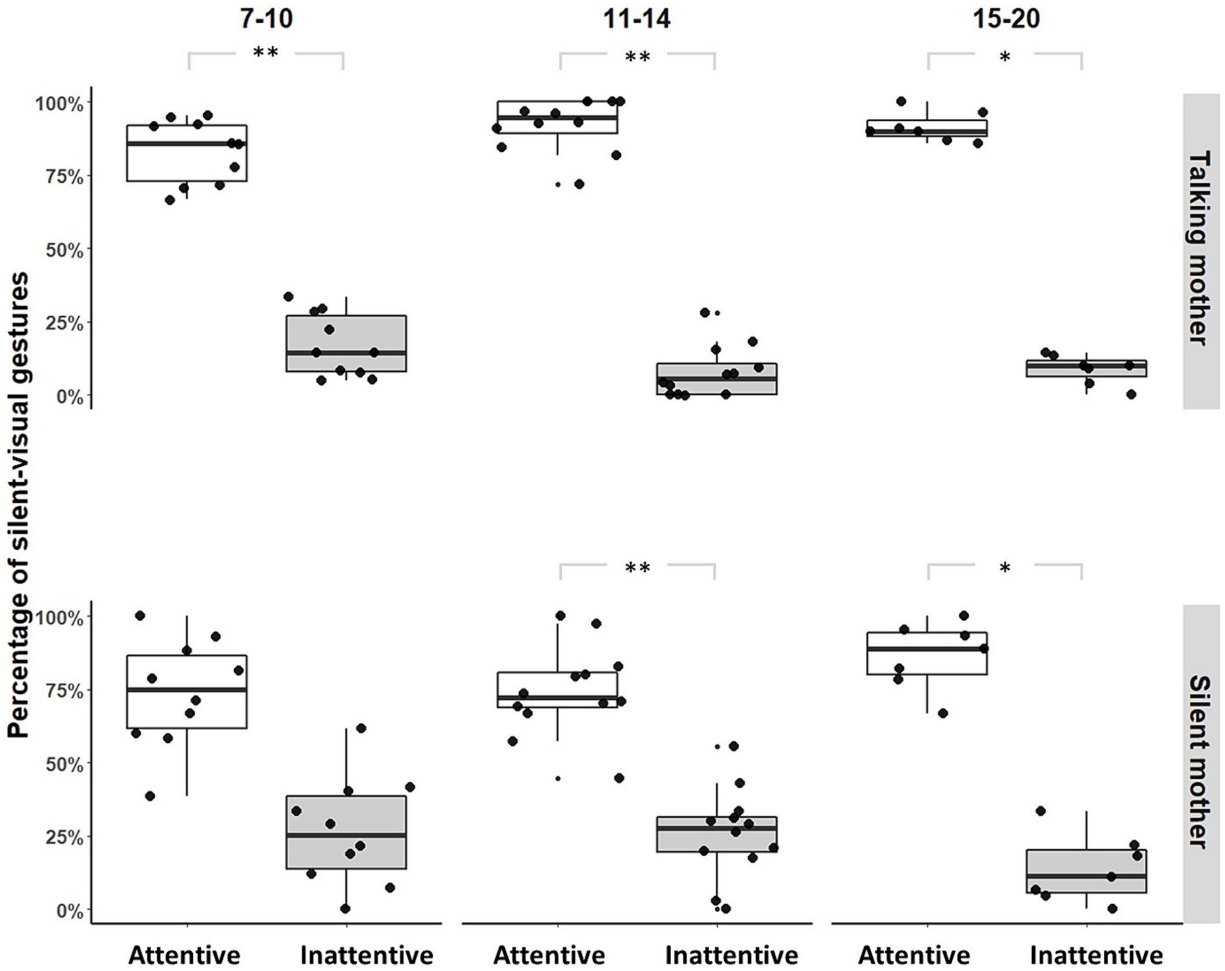
Distribution of silent-visual gestures produced by maternal condition and age range (*p-value<0.05; **p-value<0.01). Large black circles represent the mean proportion per subject. Median (horizontal lines), quartiles (boxes), percentiles (2.5 and 97.5%), vertical lines, and outliers (small black circles) are indicated. *Silent mother* refers to the condition without IDS; *Talking mother* refers to the condition with IDS.

With a talking mother (IDS), infants produced fewer silent-visual signals when the mothers did not show visual attention than when they did, regardless of age group ([7–10]: Permutation Test, *p* = 0.006, *N* = 11; [11–14]: Permutation Test, *p* = 0.001, *N* = 12; [15–20]: Permutation Test, *p* = 0.047, *N* = 7; see Figure 1). With a silent mother (No-IDS), this same pattern was found only from [11–14] months of age ([7–10]: Permutation Test, *p* = 0.076, *N* = 11; [11–14]: Permutation Test, *p* = 0.003, *N* = 12; [15–20]: Permutation Test, *p* = 0.047, *N* = 7; see Figure 1). *Unimodal* adjustment was significantly better when the mother was talking than when the mother was silent in the [7–10, 11–14] month age groups ([7–10]: Fisher’s exact tests, OR = 2.452, *p* < 0.001, *N* = 11; [11–14]: Fisher’s exact tests, OR = 3.154, *p* < 0.001, *N* = 12; [15–20]: Fisher’s exact tests, OR = 1.092, *p* = 1, *N* = 7, Supplementary Figure S1).

**Figure 2:**
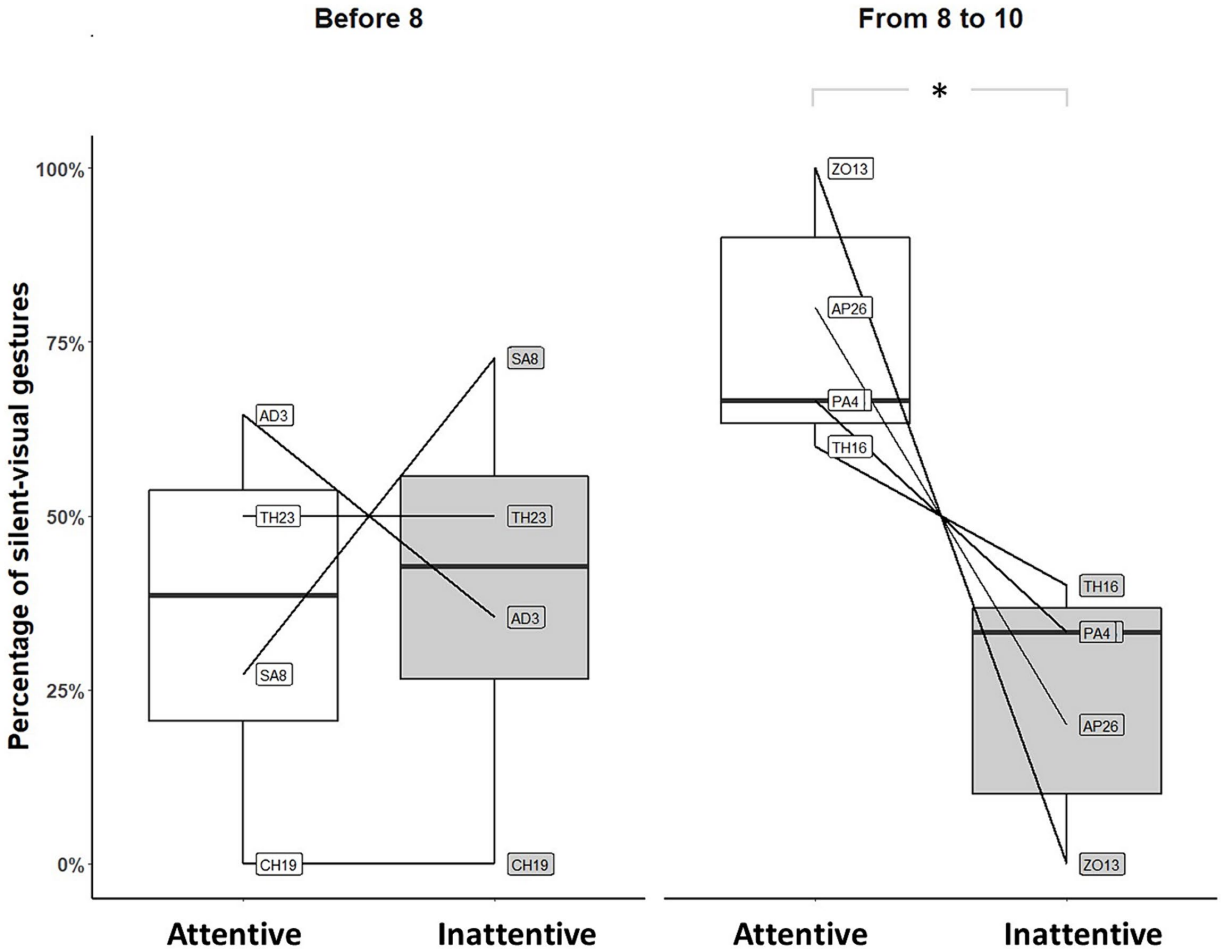
Distribution of silent-visual gestures produced by individuals before and after 8 months of age in front of silent mothers (*p-value<0.05). Large black circles represent the mean proportion per subject. Median (horizontal lines), quartiles (boxes), percentiles (2.5 and 97.5%), vertical lines, and outliers (small black circles) are indicated. *Data from the first observation only are represented. Infants not exposed to attentive and inattentive mothers during the first observation were removed from the analysis.*

We suspected that infants aged [7–10] months may constitute a heterogeneous group regarding *unimodal* adjustment. We therefore looked separately at infants younger than 8 months and those older than 8 months. Significantly fewer silent-visual signals were produced when the mothers did not show visual attention than when they did only by infants older than 8 months (before 8: Permutation Test, *p* = 1, *N* = 4; from 8 to 10: Permutation Test, *p* = 0.031, *N* = 5; see Figure 2).

### Cross-modal adjustment: how do signal modalities vary according to maternal visual attention and IDS?

**Figure 3:**
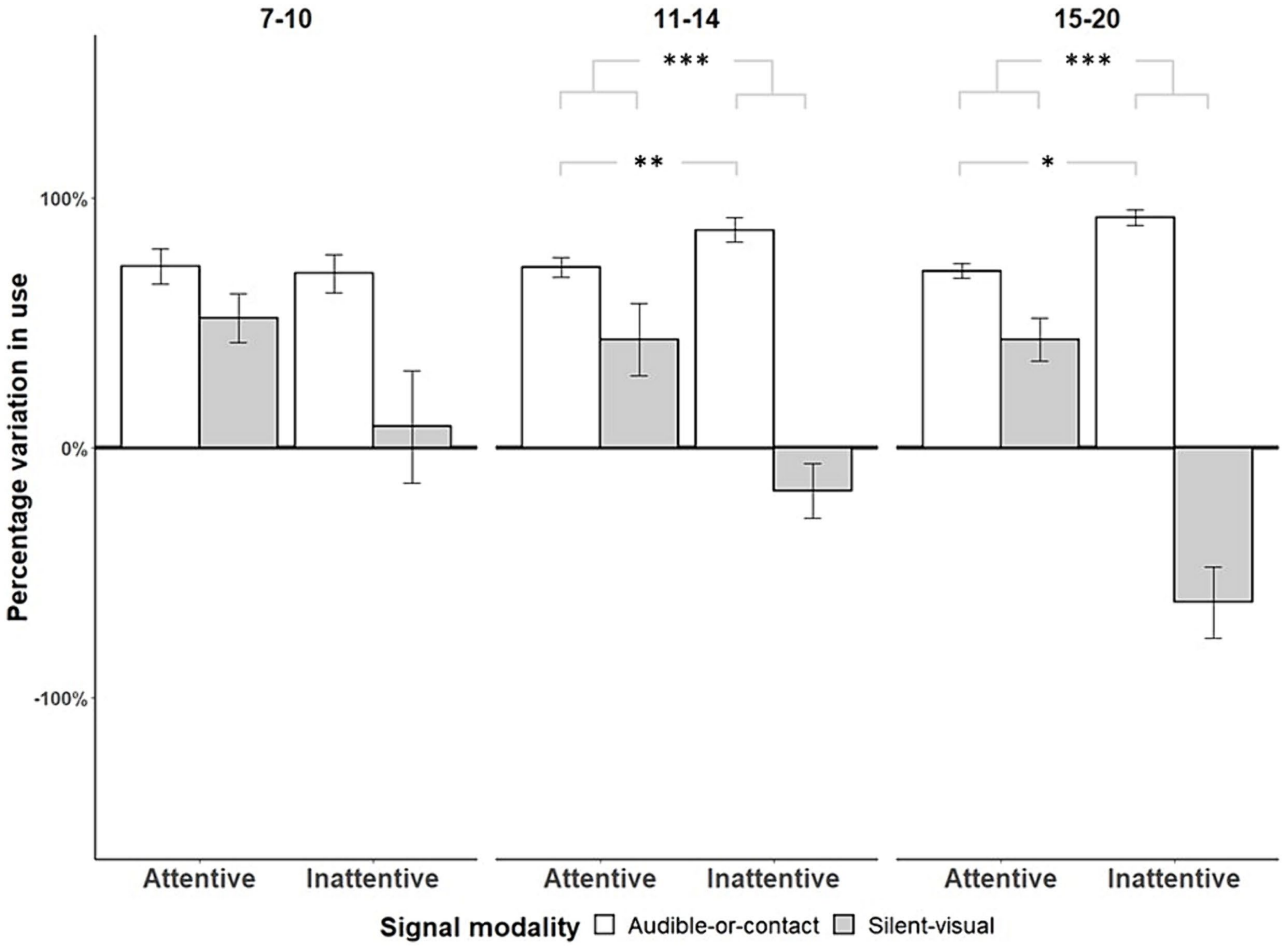
Variation in use of audible-or-contact and silent-visual gestures with respect to maternal visual attention by age range when the mothers were silent (*N* = 11, *p < 0.05, ***p < 0.001). The deviations above and below the zero-line show changes (plus standard error bar) in the use of each modality, according to the maternal state of attention before signalling, from the overall average use of that modality in signalling. *To represent active adjustment of the different gesture modalities towards the maternal visual attention state, we calculated the percentage deviation in the* var*iation in the use of audible-or-contact as compared with silent-visual gestures for each condition of maternal attention and age category (as* Hobaiter and Byrne, 2011*). The deviation was calculated by (β/α − 1) × 100 with α = number of audible-or-contact signals/total number of signals used in the age-range subgroup, and β = number of audible-or-contact signals /total number of signals used in the condition and age-range subgroup.*

Only infants of [11–14, 15–20] months of age showed capacities of *cross-modal* adjustment by producing significantly more audible-or-contact signals than silent-visual signals when the mothers did not show visual attention as compared to when they did, whether the mother was talking ([7–10]: Fisher’s exact tests, OR = 0.955, *p* = 1, *N* = 11; [11–14]: Fisher’s exact tests, OR = 0.543, *p* = 0.007, *N* = 12; [15–20]: Fisher’s exact tests, OR = 0.554, *p* = 0.036, *N* = 7; see Figure 3) or silent ([7–10]: Fisher’s exact tests, OR = 0.786, *p* = 0.348, *N* = 11; [11–14]: Fisher’s exact tests, OR = 0.524, *p* < 0.001, *N* = 12; [15–20]: Fisher’s exact tests, OR = 0.168, *p* < 0.001, *N* = 7; see Figure 3). To ascertain that these differences were not only due to the decrease in silent-visual signals in case of visual inattention (i.e., *unimodal* adjustment, see above), we tested the increased proportions of audible-or-contact signals addressed to a visually inattentive mother compared to an attentive mother using one-tailed permutation tests. When the mother was talking, infants of [11–14] months of age produced more audible-or-contact signals towards their mother when she was visually inattentive compared to when she was attentive, and a similar trend was found at [15–20] months of age ([7–10]: Permutation Test, *p* = 1, *N* = 11; [11–14]: Permutation Test, *p* < 0.001, *N* = 12; [15–20]: Permutation Test, *p* = 0.094, *N* = 7). When the mother remained silent, we found the same pattern from 11 months onwards ([7–10]: Permutation Test, *p* = 0.275, *N* = 11; [11–14]: Permutation Test, *p* = 0.006, *N* = 12; [15–20]: Permutation Test, *p* = 0.023, *N* = 7). *Cross-modal* adjustment was better when the mother was silent than when she was talking, regardless of the age group ([7–10]: Fisher’s exact tests, OR = 0.454, *p* < 0.001, *N* = 11; [11–14]: Fisher’s exact tests, OR = 0.285, *p* < 0.001, *N* = 12; [15–20]: Fisher’s exact tests, OR = 0.084, *p* < 0.001, *N* = 7).

### Do infants prefer using gestures or oral sounds when maternal visual attention is not available?

Infants produced more audible than contact signals when the mothers did not show visual attention regardless of their age and whether they faced a talking mother ([7–10]: Permutation Test, *p* = 0.006, *N* = 11; [11–14]: Permutation Test, *p* = 0.001, *N* = 12; [15–20]: Permutation Test, *p* = 0.047, *N* = 7) or a silent mother ([7–10]: Permutation Test, *p* = 0.006, *N* = 11; [11–14]: Permutation Test, *p* = 0.001, *N* = 12; [15–20]: Permutation Test, *p* = 0.047, *N* = 7).

**Figure 4:**
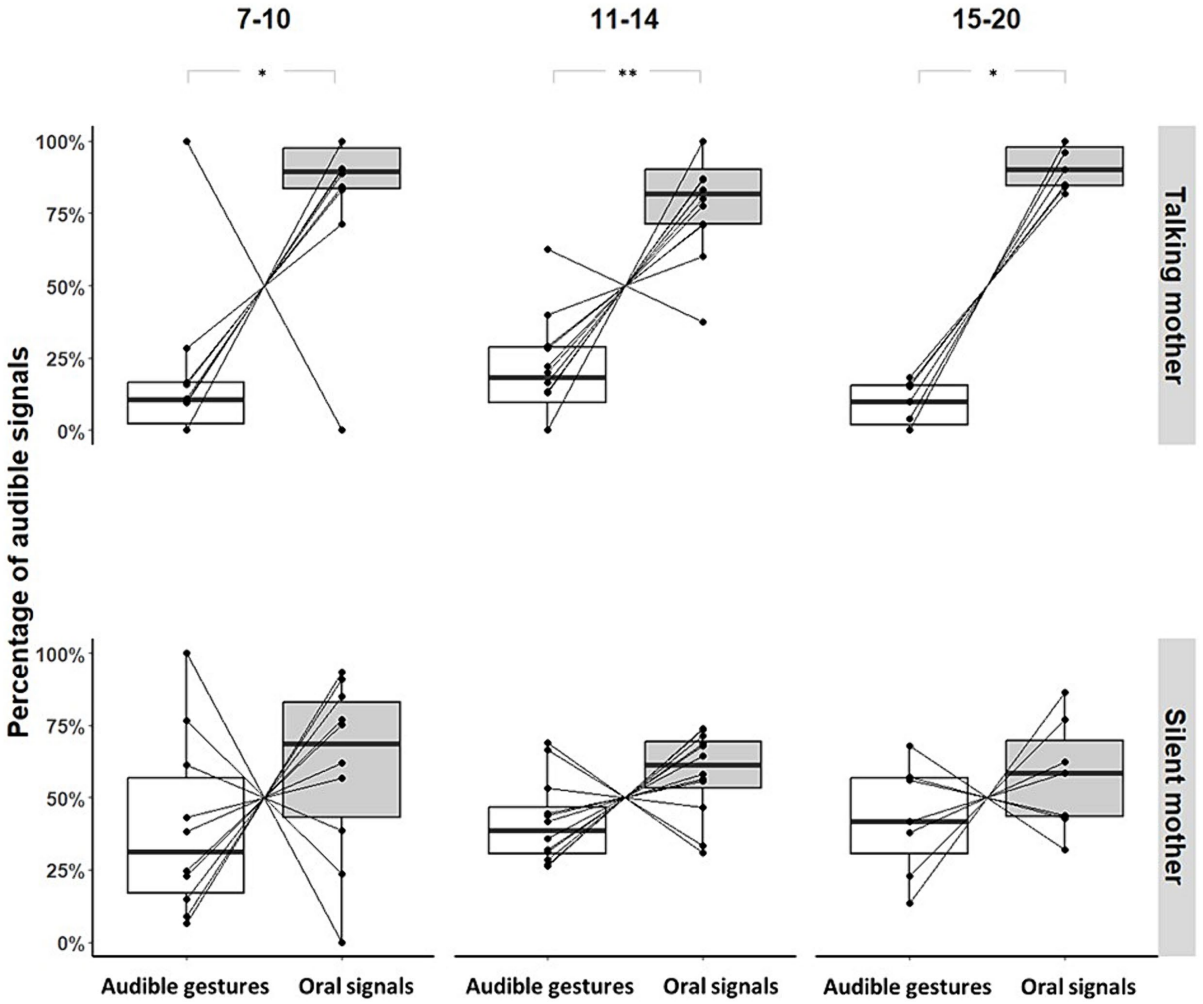
Proportions of audible gestures and oral signals produced by individual and age range when the mothers did not show visual attention (*p-value<0.05; **p-value<0.01). Large black circles represent the mean proportion per subject. Median (horizontal lines), quartiles (boxes), percentiles (2.5 and 97.5%), vertical lines, and outliers (small black circles) are indicated. *Silent mother* refers to the condition without IDS; *Talking mother* refers to the condition with IDS.

Infants facing a talking mother produced more oral than gestural audible signals when the mothers did not show visual attention; there was no age effect ([7–10]: Permutation Test, *p* = 0.035, *N* = 11; [11–14]: Permutation Test, *p* = 0.006, *N* = 12; [15–20]: Permutation Test, *p* = 0.047, *N* = 7; see Figure 4). This pattern did not reach significance for infants facing a silent mother (No-IDS; [7–10]: Permutation Test, *p* = 0.469, *N* = 11; [11–14]: Permutation Test, *p* = 0.195, *N* = 12; [15–20]: Permutation Test, *p* = 1, N = 7; see Figure 4).

### How are multimodal combinations used according to maternal visual attention and IDS?

**Figure 5:**
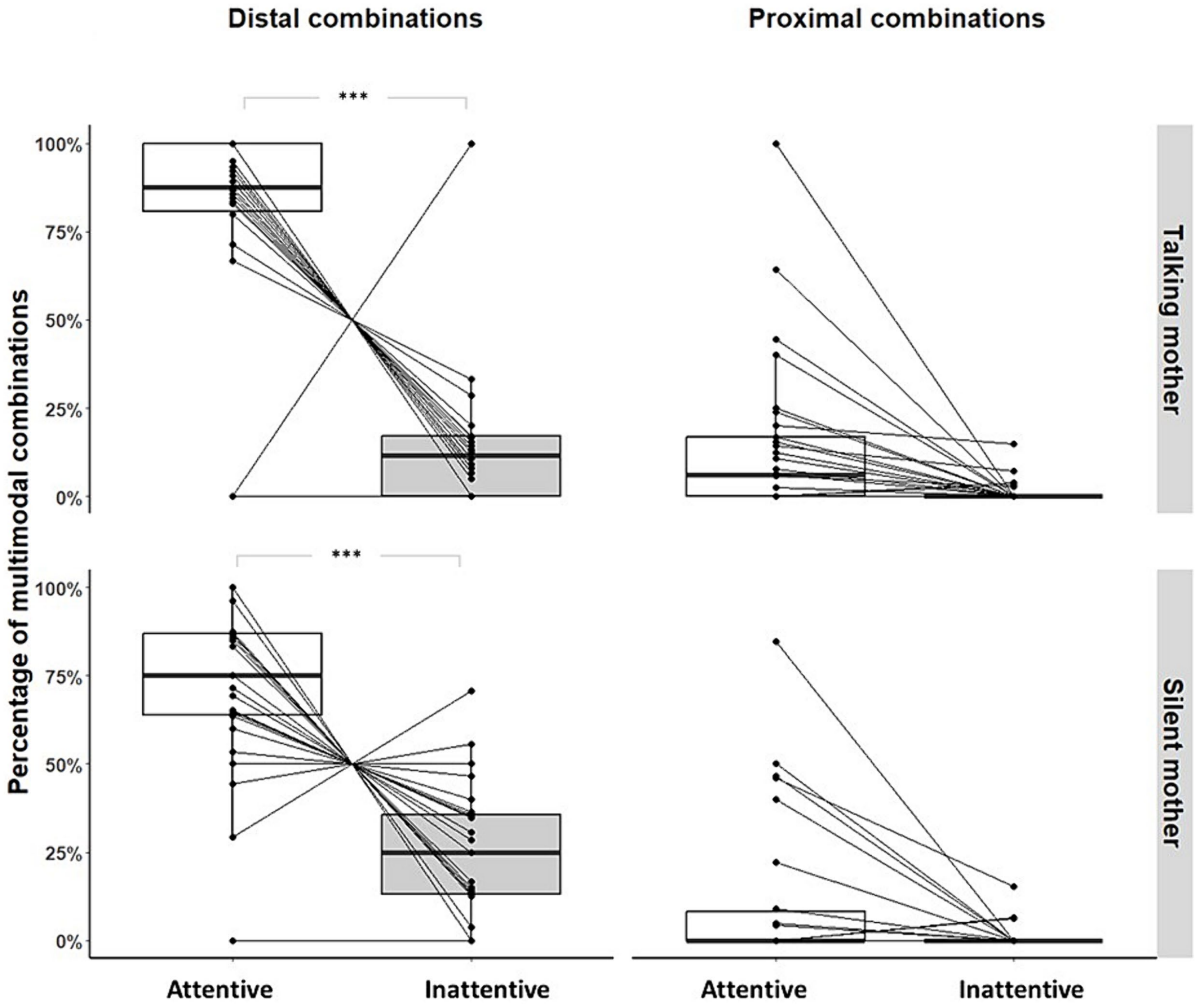
Distribution of *distal* and *proximal* multimodal combinations produced by individual and maternal visual condition (*p-value<0.05; **p-value<0.01; ***p-value<0.001). Large black circles represent the mean proportion per subject. Median (horizontal lines), quartiles (boxes), percentiles (2.5 and 97.5%), vertical lines, and outliers (small black circles) are indicated. *Silent mother* refers to the condition without IDS; *Talking mother* refers to the condition with IDS.

Within the 987 multimodal combinations coded, regardless of the maternal condition and age range, infants produced more *distal* than *proximal* combinations (distal combinations *n* = 883, 89.46%; for more details, see Table 3). Infants produced significantly more *distal combinations* when the mothers showed visual attention than when they did not, whether the mother was talking (Permutation test, *p* < 0.001; see Figure 5) or silent (Permutation test, *p* < 0.001; see Figure 5). We did not find any age effect on this pattern (talking mother: Fisher’s exact tests, *p* = 0.277, *N* = 30; silent mother: Fisher’s exact tests, *p* = 0.525, *N* = 30). Infants produced *proximal combinations* independently from maternal visual condition and IDS (talking mother: Permutation test, *p* = 0.904, *N* = 30; silent mother: Permutation test, *p* = 1, *N* = 30; see Figure 5). We did not find any age effect on this pattern (talking mother: Fisher’s exact tests, *p* = 0.451, *N* = 30; silent mother: Fisher’s exact tests, p = 1, *N* = 30). In addition, maternal IDS did not affect the *proximal combinations* (Fisher Test, OR = 0.856, *p* = 1, *N* = 30) produced by infants. However, we found a non-significant trend for the *distal combinations* (Fisher’s exact tests, OR = 2.439, *p* = 0.061, *N* = 30; Figure 5).

**Table 3:** Amount of multimodal combinations by age range and maternal attention condition.

	Maternal visual attention	Attentive				Inattentive			
	**Main category**	**Distal**	**Proximal**			**Distal**	**Proximal**		
	**Combined modalities**	**AV**	**AC**	**AVC**	**VC**	**AV**	**AC**	**AVC**	**VC**
Talking mother	[7–10]	96	11	1	6	20	0	1	0
	[11–14]	155	12	2	7	18	4	1	0
	[15–20]	164	4	2	5	18	1	0	0
	*Subtotal*	*415*	*27*	*5*	*18*	*56*	*5*	*2*	*0*
	***Global total***	***415***		***50***		***56***		***7***	
Silent mother	[7–10]	111	13	1	2	47	2	0	0
	[11–14]	120	4	1	1	38	1	0	0
	[15–20]	76	10	5	5	20	2	0	0
	*Subtotal*	*307*	*27*	*7*	*8*	*105*	*5*	*0*	*0*
	***Global total***	***307***		***42***		***105***		***5***	
	***Overall total***	***722***		***92***		***161***		***12***	

The different categories of multimodal combinations are mutually exclusive. ‘Combined modalities’ indicates the different sensory modalities included in the signal combination: ‘A’ refers to one or more audible signals, ‘V’ refers to one or more silent-visual gestures and ‘C’ refers to one or more tactile gestures.

## Discussion

This study examined the onset and development of *attention-sensitive signalling* in 7- to 20-month-old infants communicating to their mother in the naturalistic context of the home. One aim of the study was to document the use of the audible-oral modality/channel across development, for its relevance to language acquisition and evolution. According to our predictions, infants showed *unimodal* and *cross-modal* adjustment of their communicative signals to their mother’s visual attention. The ability to inhibit silent-visual signals towards visually inattentive mothers (*unimodal* adjustment) predated the ability to deploy audible-or-contact signals in this case (*cross-modal* adjustment). We also predicted that maternal infant-directed speech (IDS) would compensate the absence of visual attention, hence decreasing the infant’s signalling rates in those cases. Our results support this prediction; *cross-modal* adjustment from visually attentive to inattentive mothers is steeper when the mother remains silent than when she is talking. Beyond that, maternal IDS was influential in various ways as it was also associated with (i) a steeper *unimodal* adjustment, (ii) the preference by infants for oral signals over audible gestures to address visually inattentive mothers, and (iii) the production of distal audio-visual combinations of signals by infants facing a visually attentive mother. Overall, breakdowns in maternal visual attention are associated with increased use of the audible-oral modality/channel by infants. Maternal attentional breakdowns and IDS appear to scaffold the infants’ use of the audible-oral modality/channel, as well as the multimodal integration of vocalisations and gestures that precedes speech onset. These findings provide compelling support to the evolutionary role of the sharing of attentional resources between parents and infants into the emergence of modern language.

As stated above, the onset of *unimodal* adjustment predated the onset of *cross-modal* adjustment. Infants were actually able to adjust their silent-visual signals to their mother’s visual attention from 8 months of age, which is earlier compared to other studies (Liszkowski et al., 2008; Igualada et al., 2015; Wu and Gros-Louis, 2017). In contrast, *cross-modal* adjustment emerged between 11 and 14 months, with infants producing more audible-or-contact signals than silent-visual signals when their mother was visually inattentive as compared to when she was attentive. This result is globally consistent with previous work on infants’ coupling of pointing gestures and vocalisations in declarative pointing tasks (Gros-Louis and Wu, 2012; Wu and Gros-Louis, 2014, 2017). The early onset of *unimodal* adjustment found here may come from the consideration of an extended repertoire of signals that can possibly be adjusted earlier than pointing gestures (e.g., *smiles*: Jones et al., 1991; Jones and Hong, 2001, 2005; *proximal deictics gestures*: Rodríguez et al., 2015; Murillo et al., 2019; *gaze-coordinated body movements*: Bourjade et al., 2023; Dafreville et al., 2024).

The developmental trajectories of *unimodal* and *cross-modal* adjustment found in this study also differ from previous observations of wild immature chimpanzees’ gestural communication. While infant chimpanzees did not show any adjustment of their gestures, juveniles showed *cross-modal* adjustment prior to the onset of *unimodal* adjustment in adolescents (Dafreville et al., 2021). Human infants and immature chimpanzees did not favour the same perceptible modality in *cross-modal* adjustment; juvenile chimpanzees used preferentially contact gestures, while adolescents preferred audible gestures. In contrast, human infants observed in the context of free play preferred contact gestures (Rodrigues et al., 2021) while this study showed that they favoured the audible modality mostly through the oral channel to address inattentive mothers. These discrepancies cannot be attributed to either species differences or to developmental differences because of a lack of evidence and many contextual/methodological variations. For example, we observed very few tactile signals and proximal combinations, but part of the observation was conducted during meal time during which infants were maintained in bouncers. This constraint upon motor activity is known to alter socio-cognitive performance as well as manual exploration of the surrounding world (e.g., Thurman and Corbetta, 2019; Bard et al., 2021). This methodological choice may have affected the onset of *cross-modal* adjustment in the present study. Further investigations are needed to clarify if tactile signalling is preferred by younger infants over audible signalling to address an inattentive mother.

Our results underline the multifaceted role of *infant-directed speech* (IDS) on the use of audible-oral signals by infants, both in early pragmatic development and in the course of human evolution (Falk, 2004; Mehr and Krasnow, 2017). Following Falk (2004) hypothesis, bipedalism caused dramatic changes to mother-infant physical proximity in preventing immature new-borns from climbing on their mother by their own will. Early human mothers may have provided vocal attention instead of physical contact, while infants may have requested attention through distal means of communication like gestures and vocalisations. The current results support this hypothesis in several ways. First, we found *cross-modal* adjustment to be steeper when the mothers remained *silent* than when they were talking. The absence of IDS in addition to visual inattention may constitute a situation of global maternal inattention that is sufficiently strong to put a strain on the learning infant. In other words, full maternal inattention may prompt infants to regain their mother’s attention by using *cross-modal* adjustment. As proposed by Falk (2004), mother’s IDS may however provide a compensatory form of attention to the infant. This is consistent with the fact that infants produced fewer audible-or-contact signals towards visually inattentive mothers when they were talking compared to when they remained silent. Although we did not control for the infant’s motives for using the audible modality, like emotional distress, volubility, arousal or the mother’s responsiveness, our results indicate that infants aged 11 months onwards can strategically use the audible component of signals that are otherwise visual to address a visually inattentive mother. This suggests that they possess some sort of knowledge about the different sensory modalities of their communicative signals.

**Figure 6:**
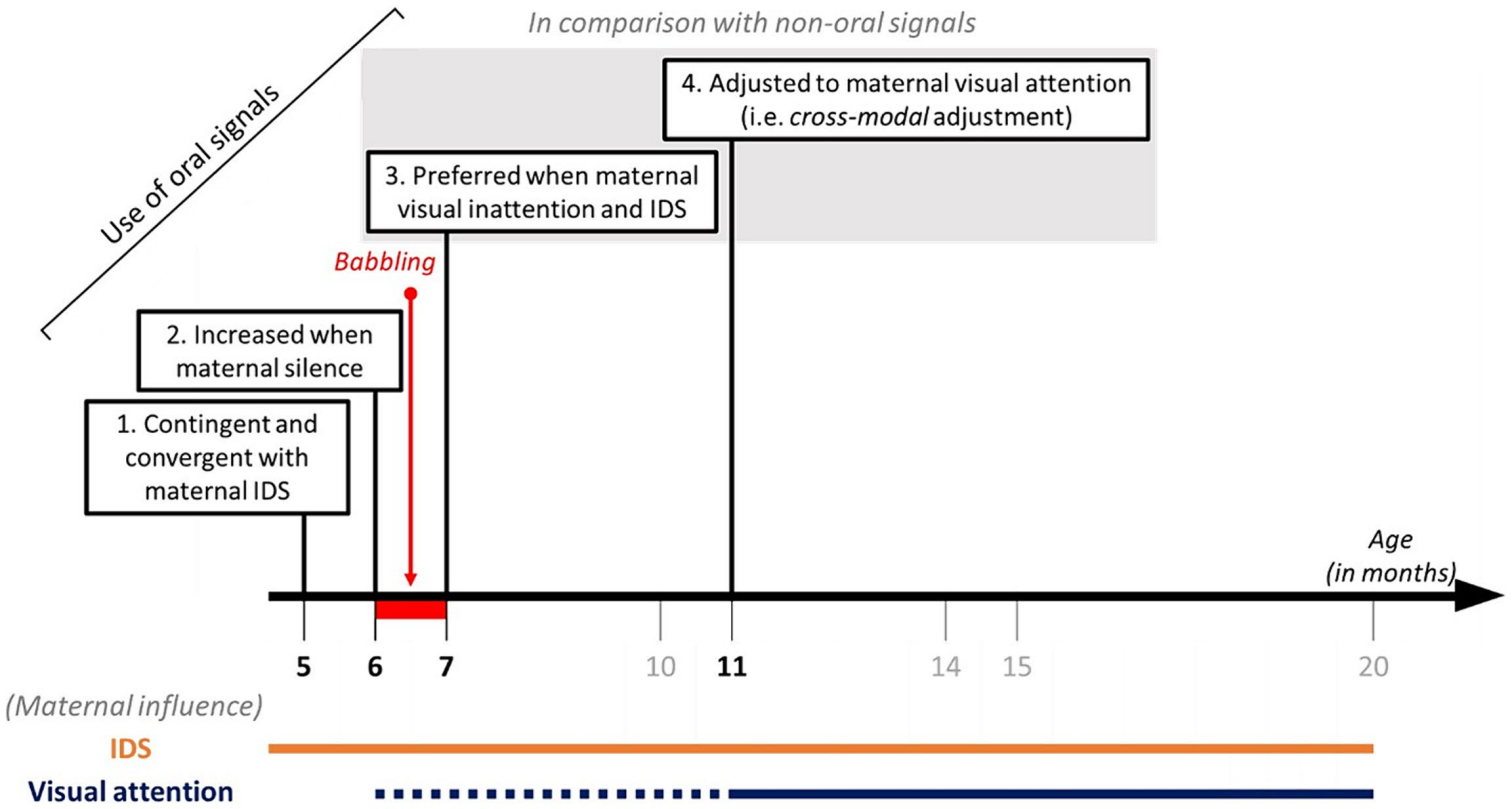
Scaffolding of the pragmatic usage of oral signals by maternal IDS and visual attention.

Many studies support the association between IDS and infant communication outcomes (Golinkoff et al., 2015; Spinelli et al., 2017; Cristia, 2022). To visualise the effect of IDS on the capacity of *unimodal* adjustment, we compared the active adjustment towards a talking mother and a silent mother during *unimodal* adjustment (see Supplementary Figure S1). Irrespective of the age category, the presence of maternal IDS prompts infants to *inhibit* their silent-visual gestures towards a visually inattentive mother rather than to produce more silent-visual gestures when she is visually attentive. A facilitation effect was also found on the production of *distal* combinations of silent-visual gestures and oral signals. Contrary to our expectations, infants did not use audio-visual combinations in *cross-modal* adjustments. Instead, they produced most audio-visual combinations towards visually attentive and talking mothers, suggesting that maternal visual engagement and IDS facilitate a multimodal combination of signals, which is concordant with other studies (e.g., coupling of vocalisations and pointing gesturesLiszkowski et al., 2008, Igualada et al., 2015, Wu and Gros-Louis, 2017; see also Fuertes et al., 2023 for broader combinations). More generally, IDS may drive the transition from *IDS-sensitive signalling* observed from 5 months of age (Delgado et al., 2002; Goldstein et al., 2009; Horvath et al., 2011; Franklin et al., 2015; Bourjade et al., 2023) to fully-fledged *attention-sensitive signalling* observed from 11 months (see Figures 4, 6). This is considered to be a building block of intentional communication in human infants and non-human animals (Townsend et al., 2017). Remarkably, the proportion of audible gestures and oral signals used in *cross-modal adjustment* varied according to the presence or absence of maternal IDS. Even if the *cross-modal* adjustment pattern was more pronounced when the mothers were silent than when they were talking, only conditions with IDS elicited a preferential use of oral signals against audible gestures. This finding supports the idea that preverbal oral signals serve more communicative functions than simply an attention-getting function. They also support the scaffolding function of IDS in the acquisition and the evolution of speech, although the prevalence of IDS differs among human populations (Cristia, 2022). Further study is needed to address attention-sensitive communication towards fathers, and more generally towards non-mother recipients, especially in eco-cultural contexts favouring cooperative caregiving (Keller, 2008).

The present study brings novel findings on the development of the voluntary use of the voice (vocal control) by human infants. Figure 6 summarises the recent evidence on this topic. Five-month-old infants already produce oral signals that are contingent and convergent in terms of acoustic parameters with the caregiver’s IDS (Whalen et al., 1995; Holowka and Petitto, 2002; DePaolis et al., 2013; Fröhlich and van Schaik, 2020; Northrup and Iverson, 2020). At 6 months of age, infants increase their production of audible signals (both gestural and oral) when the mothers are silent, regardless of their visual attention state (Delgado et al., 2002; Bourjade et al., 2023). The current results suggest two additional steps in the development of vocal control and its integration into an extended multimodal communication system. At 7 months, infants preferentially use oral sounds over audible gestures to address a visually inattentive mother who is talking to them (Figures 3, 4, 6). Then, 11-month-old infants are truly capable of *cross-modal adjustment*, based on the global preference for oral signals to address visually inattentive mothers who are talking to them (Figures 4, 6). These findings contribute important knowledge on the developmental acquisition of pragmatic skills that rely on voluntary control of the voice. Visual breakdowns from the mother, together with her infant-directed speech appear to scaffold an infant’s capabilities for controlling oral signals, including voluntary usage of the voice.

## Conclusion

This study was one of the first to give a comprehensive overview of the development of *attention-sensitive signalling*, a keystone pragmatic skill for language acquisition by human children. The naturalistic and multimodal study design provides important knowledge about the early onset of unimodal and cross-modal adjustments between 7 and 11 months of age. Most importantly, maternal scaffolding of infants’ early pragmatic skills may depend on the disruption of her visual attention and infant-directed speech. The audible channel is favoured by infants in cases of visual inattention, while the oral channel, including voiced and non-voiced sounds is favoured when visually inattentive mothers are talking to their child. In contrast, infants favour distal audio-visual combinations when their mother is visually attentive and talking. Further study of the acoustic parameters of these developmental precursors of speech may help to disambiguate their communicative functions (Pisanski et al., 2016). Overall, these findings support the hypothesis that vocal control emerges within the sharing of attentional resources between mothers and infants.

## Data availability statement

The raw data supporting the conclusions of this article will be made available by the authors, without undue reservation.

## Ethics statement

The studies involving humans were approved by Ethical committee of the University of Toulouse (i.e., CER-NI; n IRB00011835-2019-03-19-141). The studies were conducted in accordance with the local legislation and institutional requirements. Written informed consent for participation in this study was provided by the participants' legal guardians/next of kin.

## Author contributions

MD: Conceptualization, Data curation, Formal analysis, Investigation, Methodology, Writing – original draft, Writing – review & editing. MG: Supervision, Writing – review & editing. MB: Conceptualization, Investigation, Supervision, Validation, Writing – review & editing.

## Footnotes

^1^http://cran.r-project.org
