# Supplementary material for: Attention-sensitive signalling by 7- to 20-month-old infants in a comparative perspective
Source: Front Psychol. 2024 Mar 18;15:1257324. doi: 10.3389/fpsyg.2024.1257324 (PMC10982422; doi:10.3389/fpsyg.2024.1257324)
Supplement: Supplementary file 1 [file Data_Sheet_1.DOCX]

**Supplementary material**

Attention-sensitive signalling by 7-to-20-month-old infants in a comparative perspective.

Mawa Dafreville^1*^, Michèle Guidetti ^1^, Marie Bourjade^1,2^

^1^ CLLE, Université de Toulouse, CNRS, Toulouse, France

^2^ Institut Universitaire de France

Table of contents

[Behavioral repertoire, inter-rater reliability and signal sampling 2](#_Toc149552923)

[Behavioral repertoire (Table S1, S2) 2](#_Toc149552924)

[Inter-rater reliability (Table S3, S4) 9](#_Toc149552925)

[Signal sampling (Table S5) 10](#_Toc149552926)

[Exploratory analyses 14](#_Toc149552927)

[a) Rationale of the statistical analyses 14](#_Toc149552928)

[b) Nuisance control of confound variables 14](#_Toc149552929)

[c) Tests of maternal attention variables (Table S6) 15](#_Toc149552930)

[Additional illustration (Figure S1) 18](#_Toc149552931)

# Behavioral repertoire, inter-rater reliability and signal sampling

## Behavioral repertoire (Table S1, S2)

**TABLE S1. Repertoire of human 7-to-20-month-old infants’ multimodal communication.**

|  |  |  |  |  |
| --- | --- | --- | --- | --- |
| **Visual gestures** |  |  | *Onset* | *Offset* |
| Body movement | Circular movement of the limbs (VAM) | Repetitive movements of the arms and/or legs and/or hands and/or fingers performed two or more times.  Included: the infant holds a silent object during it; when the infant swings his/her arms while walking.  Excluded : the infant holds a noisy object during it (code AO) | It starts at the image on which a movement is visible. | A new occurrence is recorded after a break longer than 1 second. |
|  | Synchronised Trunk/Members movement (VSTM) | The infant leans back against the chair or against his/her mother and raises the pelvis up while stretching the trunk and the legs. The arms can be raised up or not.  Included: when the infant is sitting on his/her mother's lap in an embrace position for example and leans back against her. | It starts when the trunk is stretching. | A new occurrence is recorded after a break longer than 1 second. |
|  | Swagger (VS) | The infant moves his/her trunk along an axis (back and forth, from side to side, or up and down). An occurrence is recorded if at least a two-way movement is achieved (e.g. one movement back followed by one movement forth). | It starts when the movement begins. | When the infant stops moving. |
| Deictic gestures | Whole-hand pointing gesture (VWHPO) | The infant points with the whole hand and fingers extended in the direction of the object or another individual. | It starts when the infant begins his/her movement towards the object/individual | It ends when the infant releases his/her arm (even a little) or changes the target |
|  | Index-pointing gesture (VIPO) | The infant points with his/her index finger towards an external object or individual (triadic pattern). | It starts when the infant begins his/her movement towards the object/individual. | It ends when the infant releases his/her arm or changes the target. |
|  | Reaching attempt (VRAO) | The infant tries to reach out for one (or more) object(s)/or another individual out of range, without pointing, but leaning the trunk towards the object in a time window of 1 second before or after stretching his/her arm with his/her hand open. The infant can repeatedly open and close his/her fingers in the direction of the object (e.g., grasping movements).  Excluded: when the infant makes the same movement but with an object in his/her hand already. | It starts when the infant begins his/her movement towards the object/individual. | It ends when the infant releases his/her arm or changes the target. |
|  | Giving (VGO) | The infant holds the object with at least one hand towards the partner or he/she makes the object accessible to the recipient. | It starts when the infant begins his/her movement towards the object. | It ends when the infant no longer has the object in his/her hand. |
|  | Receiving (VRO) | The infant takes/touches, with at least one of his/her hands, the object that the partner has handed or made accessible to him/her.  Included: when the infant holds food that is not intended for him/her (e.g. when the partner is eating).  Excluded: when it is included in the feeding (for example when the mother gives some pasta) if the infant was not holding it before. | It starts when the infant touches the object that the partner is handing to him/her. | It ends 1 second after the first contact between the infant and the object. |
|  | Showing (VSO) | The infant without outstretched arm, holds in his/her hands an object in the direction of the partner, looking at the partner for more than 1 second while keeping the posture.  Included: pointing towards himself/herself or the partner’s body (dyadic pattern). For example, when the mother asks him/her to show her a part of his/her body (“where’s your nose?”). | It starts when the child looks at his/her partner with the object in his/her hands. | It ends when the child relaxes his/her arms or changes posture. |
| Conventional Gesture | Conventional Gesture (CVG) | The infant performs a conventional gesture, i.e. one that requires some forms of common ground to be understood.  Included: yes-no with the head; Hi with the hand; bravo with his/her hands; Gestures from an ontogenetic ritualization (e.g. when the infant reaches both arms upwards towards his/her communication partner to be carried aloft by the partner or the infant hides his/her face to play *PeekaBoo*); We also included gestures from sign language; Also, if the infant touches his mother’s breast with his/her hands and the mother makes a reference to breastfeeding. | It starts when the infant begins the conventional gesture. | It ends when the infant stops the movement during more than 1 second; a new occurrence is recorded after this interval. |
| Facial Expression | Facial Expression- Smiling (FX) | Any movement of the mouth likened to a smile, that results in an increased distance between the two corners of the mouth and any movement of the eyebrows that rise while looking at his/her mother, father or siblings. | It starts when the infant begins producing facial expression (the infant starts smiling) while looking at his/her partner. | A new occurrence is recorded after 1 second spent without smiling or looking at his/her partner. |
| **Tactile gestures** |  |  | *Onset* | *Offset* |
| Touching | Physical contact (TM) | The infant punctually engages a gesture with a part of his/her body (exclusively hands or head) that leads to physical contact with his/her mother (e.g. grab, bite, push, clap…). This has to last less than 5 seconds. If there are repetitive contacts less than 1 second apart by the same body part (for example the right hand), it is considered the same occurrence. If the contact made by the baby lasts more than a second, the behaviour is automatically coded as a behaviour lasting 1 second (segment of 1 second on ELAN).  Included: when the infant touches his/her mother's breast (referring to breastfeeding VCG); When the infant touches his/her mother's clothes while embracing her (e.g. the infant grabs his/her mother dress).  Excluded: when the infant is touching his/her mother because her/his mother is embracing or carrying him/her (indirectly touching);  Also when the infant is touching the mother's breast while breastfeeding  (except if he/she is touching his/her mother’s face or hands); When the infant is touching his/her mother with an object. | It starts when an infant’s body part touches his/her mother. | It ends when there is no physical contact between the infant and the mother or if the baby changes parts of body in contact. |
| **Audible Signals** |  |  | *Onset* | *Offset* |
| Non-voiced sounds | Mouth sounds (VOMS) | Any non-voiced sound resulting from the air breathed in and out, without vibrating the vocal folds (e.g. sigh, tongue clicking, raspberry).  Included: loud breathing sound that lasts less than 5 seconds.  Excluded: if the infant has food in his/her mouth while producing mouth sounds (e.g. while breastfeeding). | It starts when the sound is first audible. | It ends only if the mother talks to her baby while the sound is stopped or after 1 second silent or if another type of oral sound replaces it (no acoustic gestures) while the sound is stopped |
|  | Laughs (VOL) | Smiles with vocal or non-vocal sounds are defined as spontaneous laughs (Kawakami et al., 2007) continuing for more than 1 second, interspersed by silences during which he/she breathes in. | It starts when the sound is audible. | It ends only if the mother talks to her baby while the sound is stopped or after 1 second silent or if another type of oral sound replaces it (no acoustic gestures) while the sound is stopped. |
| Voiced sounds | Vowel-like sounds (VOP) | Vowel-like sounds due to the vibration of the vocal folds, as well as squeals and growls.  Excluded: vegetative sounds (e.g., sneezes or burps). | It starts when the sound is first audible. | It ends only if the mother talks to her baby while the sound is stopped or after 1 second silent or if another type of oral sound replaces it (no acoustic gestures) while the sound is stopped. |
|  | Grunt (VOG) | The infant produces a vowel sound on breathing out with a rhythmic pattern where the impulse is on a strong beat and the resulting sound on a weak beat. | It starts when the sound is audible. | It ends only if the mother talks to her baby while the sound is stopped or after 1 second silent or if another type of oral sound replaces it (no acoustic gestures) while the sound is stopped. |
|  | Babbling (VOB) | The infant emits short or continuous sounds, composed of vowels and consonants forming isolated or repeated syllables, from the vibration of the vocal folds and the opening and closing of the mouth. | It starts when the sound is audible. | It ends only if the mother talks to her baby while the sound is stopped or after 1 second silent or if another type of oral sound replaces it (no acoustic gestures) while the sound is stopped. |
|  | Cries (VOCR) | The infant utters a continuous high-pitched expiratory sound intertwined with inspiratory sounds that can be voiced or not and accompanied by tears or not. | It starts when the sound is first audible. | It ends only if the mother talks to her baby while the sound is stopped or after 1 second silent or if another type of oral sound replaces it (no acoustic gestures) while the sound is stopped |
|  | Pseudo-word (VONW) | The infant does not clearly say a word but utters a word which sounds like it. We recorded it only when the parent repeated the word to confirm it (e.g. when the child said, "Caaaaag" and his/her parent needs to confirm by asking him/her, "what do you want? Do you want some cake?”). | It starts when the infant pronounces the first syllable of the pseudo-word. | It ends at the end of the pseudo-word. |
|  | Word (VOW) | The infant says a single word of one of his/her parent languages that the parent understands. | It starts when the infant pronounces the first syllable of the word. | It ends at the end of the word. |
|  | Word succession (VOWS) | The infant clearly says a clause comprising at least subject and an object that the parent understands. | It starts when the child pronounces the first syllable of the first word of the clause. | A new clause is recorded for each association of subject, verb and object. |
| Audible gestures | Sound made with an object (AO) | Any sound produced by an action in the environment with an object in the hands (e.g., hit, scratch, scrape).  Included: if the infant pulls on a tree branch that makes a noise.  Excluded: Oral sounds or if the sound results in a movement made by the mother (for example, she grasps her baby’s arm in which he/she holds a maracas); Sounds made by an electronic toy. | It starts when the sound is audible. | It ends only if the mother talks to her baby while the sound is stopped or after 1 second silent or if another type of acoustic gesture replaces it (excluded : non-voiced sounds and vocalizations) while the sound is stopped. |
|  | Sound made without an object (AWO) | Any sound produced by an action on the environment without an object (e.g., hit, scratch, scrape) or by a self-directed action (e.g., clap hands) and the baby is not moving in the room.  Included: noise stirring the water (i.e. playing in a stream) Excluded: oral sounds | It starts when the sound is audible. | It ends only if the mother talks to her baby while the sound is stopped or after 1 second silent or if another type of acoustic gesture replaces it (excluded : non-voiced sounds and vocalizations) while the sound is stopped. |

**Table S2. Maternal attention variables used in the study**

| **Maternal attention variables** | **Definitions** |
| --- | --- |
| Physical contact | The mother has one or several body parts in physical contact with her infant. |
| Visual attention | The mother's head is directed towards her infant, the geometric lines of sight of the two individuals can cross or the infant is in the mother's line of sight. Visual attention is not considered if the mother has her eyes closed. |
| Auditory attention | The mother is sufficiently close to hear her infant vocalising. As the auditory perception of the mother cannot be measured, auditory attention was systematically considered as available.  Warning:  If the mother uses a food processor or other noisy equipment, please provide the information in comments  When the mother is out of sight, auditory attention was considered as available. |
| IDS | The mother is talking to her infant while interacting with her infant. Included: interjections (oh lala, hop, ah ahhh)  Included: mouth sounds, interjections, onomatopoeia, babbling or protophone imitation.  Excluded: mother’s laughs. |

## Inter-rater reliability (Table S3, S4)

**Table S3. Summary of Cohen’s Kappa coefficients for each infant’s communication modality**

| **Communicative modality** | **Infant’s behaviour** | **Cohen’s Kappa coefficient** |
| --- | --- | --- |
| Audible | Acoustic gestures | 0.83 |
|  | Non-voiced sounds | 0.72 |
|  | Vocalisations |  |
| Silent-visual | Body movements | 0.75 |
|  | Conventional gestures |  |
|  | Facial expression |  |
|  | Deictic gestures |  |
| Tactile | Touch* |  |

***** As tactile gestures were quite rare in the final database (in comparison to the other signals), we included them in the calculation of Cohen’s Kappa coefficient of silent-visual gestures.

**Table S4. Summary of Cohen’s Kappa coefficients for each mother’s attention category**

| **Mother’s attention state** | **Cohen’s Kappa coefficient** |
| --- | --- |
| Visual and tactile (in)attention* | 0.89 |
| IDS | 0.81 |

***** As the auditory perception of the mother cannot be measured, auditory attention was systematically considered as equal to 1.

## Signal sampling (Table S5)

**Table S5. Number of signals collected by infant per given signal category, maternal visual attention, infant-directed speech and age group.**

| ID | Signals | Talking | | Total with talking mother | Silent | | Total with silent mother | **Global total** |
| --- | --- | --- | --- | --- | --- | --- | --- | --- |
|  |  | Attentive | Inattentive |  | Attentive | Inattentive |  |  |
| **[7-10]** |  | **670** | **125** | **795** | **1126** | **613** | **1739** | **2534** |
| AD3 |  | 63 | 5 | 68 | 144 | 106 | 250 | 318 |
|  | Audible | 40 | 4 | 44 | 104 | 93 | 197 | 241 |
|  | Tactile | 5 | 0 | 5 | 8 | 0 | 8 | 13 |
|  | Visual | 18 | 1 | 19 | 32 | 13 | 45 | 64 |
| AL30 |  | 102 | 8 | 110 | 123 | 61 | 184 | 294 |
|  | Audible | 91 | 7 | 98 | 110 | 58 | 168 | 266 |
|  | Tactile | 0 | 0 | 0 | 0 | 0 | 0 | 0 |
|  | Visual | 11 | 1 | 12 | 13 | 3 | 16 | 28 |
| AP26 |  | 99 | 13 | 112 | 130 | 108 | 238 | 350 |
|  | Audible | 67 | 9 | 76 | 102 | 105 | 207 | 283 |
|  | Tactile | 8 | 0 | 8 | 13 | 1 | 14 | 22 |
|  | Visual | 24 | 4 | 28 | 15 | 2 | 17 | 45 |
| CH19 |  | 10 | 11 | 21 | 10 | 1 | 11 | 32 |
|  | Audible | 10 | 10 | 20 | 7 | 1 | 8 | 28 |
|  | Tactile | 0 | 1 | 1 | 3 | 0 | 3 | 4 |
|  | Visual | 0 | 0 | 0 | 0 | 0 | 0 | 0 |
| CL7 |  | 117 | 14 | 131 | 230 | 54 | 284 | 415 |
|  | Audible | 82 | 12 | 94 | 190 | 52 | 242 | 336 |
|  | Tactile | 11 | 0 | 11 | 14 | 0 | 14 | 25 |
|  | Visual | 24 | 2 | 26 | 26 | 2 | 28 | 54 |
| EL15 |  | 78 | 12 | 90 | 169 | 29 | 198 | 288 |
|  | Audible | 52 | 7 | 59 | 132 | 20 | 152 | 211 |
|  | Tactile | 2 | 1 | 3 | 4 | 0 | 4 | 7 |
|  | Visual | 24 | 4 | 28 | 33 | 9 | 42 | 70 |
| PA4 |  | 63 | 23 | 86 | 106 | 36 | 142 | 228 |
|  | Audible | 58 | 21 | 79 | 96 | 33 | 129 | 208 |
|  | Tactile | 1 | 0 | 1 | 4 | 0 | 4 | 5 |
|  | Visual | 4 | 2 | 6 | 6 | 3 | 9 | 15 |
| SA8 |  | 16 | 6 | 22 | 69 | 139 | 208 | 230 |
|  | Audible | 9 | 4 | 13 | 48 | 101 | 149 | 162 |
|  | Tactile | 2 | 0 | 2 | 1 | 6 | 7 | 9 |
|  | Visual | 5 | 2 | 7 | 20 | 32 | 52 | 59 |
| TH16 |  | 57 | 29 | 86 | 25 | 23 | 48 | 134 |
|  | Audible | 33 | 19 | 52 | 13 | 15 | 28 | 80 |
|  | Tactile | 0 | 0 | 0 | 0 | 0 | 0 | 0 |
|  | Visual | 24 | 10 | 34 | 12 | 8 | 20 | 54 |
| TH23 |  | 17 | 3 | 20 | 71 | 56 | 127 | 147 |
|  | Audible | 9 | 1 | 10 | 64 | 51 | 115 | 125 |
|  | Tactile | 1 | 0 | 1 | 0 | 0 | 0 | 1 |
|  | Visual | 7 | 2 | 9 | 7 | 5 | 12 | 21 |
| ZO13 |  | 48 | 1 | 49 | 49 | 0 | 49 | 98 |
|  | Audible | 28 | 0 | 28 | 39 | 0 | 39 | 67 |
|  | Tactile | 0 | 0 | 0 | 0 | 0 | 0 | 0 |
|  | Visual | 20 | 1 | 21 | 10 | 0 | 10 | 31 |
| **[11-14]** | **11-14** | **1317** | **205** | **1522** | **1296** | **690** | **1986** | **3508** |
| AI23 |  | 68 | 7 | 75 | 77 | 34 | 111 | 186 |
|  | Audible | 39 | 5 | 44 | 58 | 30 | 88 | 132 |
|  | Tactile | 2 | 0 | 2 | 0 | 0 | 0 | 2 |
|  | Visual | 27 | 2 | 29 | 19 | 4 | 23 | 52 |
| AL20 |  | 173 | 12 | 185 | 116 | 9 | 125 | 310 |
|  | Audible | 130 | 12 | 142 | 98 | 9 | 107 | 249 |
|  | Tactile | 1 | 0 | 1 | 0 | 0 | 0 | 1 |
|  | Visual | 42 | 0 | 42 | 18 | 0 | 18 | 60 |
| AL24 |  | 30 | 2 | 32 | 174 | 10 | 184 | 216 |
|  | Audible | 23 | 2 | 25 | 137 | 9 | 146 | 171 |
|  | Tactile | 0 | 0 | 0 | 0 | 0 | 0 | 0 |
|  | Visual | 7 | 0 | 7 | 37 | 1 | 38 | 45 |
| AR11 |  | 108 | 10 | 118 | 65 | 24 | 89 | 207 |
|  | Audible | 75 | 8 | 83 | 45 | 19 | 64 | 147 |
|  | Tactile | 2 | 1 | 3 | 1 | 0 | 1 | 4 |
|  | Visual | 31 | 1 | 32 | 19 | 5 | 24 | 56 |
| AX15 |  | 137 | 36 | 173 | 74 | 59 | 133 | 306 |
|  | Audible | 109 | 30 | 139 | 57 | 50 | 107 | 246 |
|  | Tactile | 3 | 4 | 7 | 0 | 2 | 2 | 9 |
|  | Visual | 25 | 2 | 27 | 17 | 7 | 24 | 51 |
| CL17 |  | 161 | 37 | 198 | 193 | 89 | 282 | 480 |
|  | Audible | 111 | 30 | 141 | 163 | 83 | 246 | 387 |
|  | Tactile | 12 | 0 | 12 | 6 | 0 | 6 | 18 |
|  | Visual | 38 | 7 | 45 | 24 | 6 | 30 | 75 |
| CO15 |  | 217 | 7 | 224 | 150 | 113 | 263 | 487 |
|  | Audible | 183 | 7 | 190 | 143 | 110 | 253 | 443 |
|  | Tactile | 4 | 0 | 4 | 0 | 0 | 0 | 4 |
|  | Visual | 30 | 0 | 30 | 7 | 3 | 10 | 40 |
| GA3 |  | 60 | 9 | 69 | 95 | 73 | 168 | 237 |
|  | Audible | 38 | 9 | 47 | 80 | 68 | 148 | 195 |
|  | Tactile | 4 | 0 | 4 | 1 | 0 | 1 | 5 |
|  | Visual | 18 | 0 | 18 | 14 | 5 | 19 | 37 |
| LA16 |  | 48 | 7 | 55 | 42 | 112 | 154 | 209 |
|  | Audible | 28 | 5 | 33 | 34 | 102 | 136 | 169 |
|  | Tactile | 0 | 0 | 0 | 0 | 0 | 0 | 0 |
|  | Visual | 20 | 2 | 22 | 8 | 10 | 18 | 40 |
| LO6 |  | 102 | 5 | 107 | 122 | 33 | 155 | 262 |
|  | Audible | 78 | 4 | 82 | 115 | 30 | 145 | 227 |
|  | Tactile | 0 | 0 | 0 | 1 | 0 | 1 | 1 |
|  | Visual | 24 | 1 | 25 | 6 | 3 | 9 | 34 |
| MA12 |  | 153 | 40 | 193 | 104 | 74 | 178 | 371 |
|  | Audible | 115 | 31 | 146 | 91 | 64 | 155 | 301 |
|  | Tactile | 2 | 1 | 3 | 1 | 1 | 2 | 5 |
|  | Visual | 36 | 8 | 44 | 12 | 9 | 21 | 65 |
| MA26 |  | 60 | 33 | 93 | 84 | 60 | 144 | 237 |
|  | Audible | 42 | 24 | 66 | 75 | 56 | 131 | 197 |
|  | Tactile | 0 | 2 | 2 | 0 | 0 | 0 | 2 |
|  | Visual | 18 | 7 | 25 | 9 | 4 | 13 | 38 |
| **[15-20]** | **15-20** | **902** | **143** | **1045** | **817** | **463** | **1280** | **2325** |
| AN13 |  | 183 | 33 | 216 | 23 | 15 | 38 | 254 |
|  | Audible | 141 | 25 | 166 | 19 | 13 | 32 | 198 |
|  | Tactile | 3 | 2 | 5 | 0 | 0 | 0 | 5 |
|  | Visual | 39 | 6 | 45 | 4 | 2 | 6 | 51 |
| AR13 |  | 113 | 11 | 124 | 138 | 39 | 177 | 301 |
|  | Audible | 70 | 7 | 77 | 110 | 37 | 147 | 224 |
|  | Tactile | 3 | 0 | 3 | 0 | 0 | 0 | 3 |
|  | Visual | 40 | 4 | 44 | 28 | 2 | 30 | 74 |
| AU30 |  | 96 | 13 | 109 | 66 | 48 | 114 | 223 |
|  | Audible | 68 | 10 | 78 | 54 | 45 | 99 | 177 |
|  | Tactile | 1 | 0 | 1 | 3 | 1 | 4 | 5 |
|  | Visual | 27 | 3 | 30 | 9 | 2 | 11 | 41 |
| LO3 |  | 101 | 16 | 117 | 138 | 30 | 168 | 285 |
|  | Audible | 66 | 11 | 77 | 110 | 28 | 138 | 215 |
|  | Tactile | 5 | 0 | 5 | 7 | 1 | 8 | 13 |
|  | Visual | 30 | 5 | 35 | 21 | 1 | 22 | 57 |
| MA25 |  | 175 | 19 | 194 | 209 | 57 | 266 | 460 |
|  | Audible | 134 | 19 | 153 | 164 | 57 | 221 | 374 |
|  | Tactile | 7 | 0 | 7 | 13 | 0 | 13 | 20 |
|  | Visual | 34 | 0 | 34 | 32 | 0 | 32 | 66 |
| NI7 |  | 159 | 39 | 198 | 67 | 97 | 164 | 362 |
|  | Audible | 121 | 33 | 154 | 59 | 96 | 155 | 309 |
|  | Tactile | 2 | 2 | 4 | 0 | 0 | 0 | 4 |
|  | Visual | 36 | 4 | 40 | 8 | 1 | 9 | 49 |
| VI13 |  | 75 | 12 | 87 | 176 | 177 | 353 | 440 |
|  | Audible | 49 | 11 | 60 | 157 | 168 | 325 | 385 |
|  | Tactile | 0 | 0 | 0 | 1 | 4 | 5 | 5 |
|  | Visual | 26 | 1 | 27 | 18 | 5 | 23 | 50 |
| **Global total** |  | **2889** | **473** | **3362** | **3239** | **1766** | **5005** | **8367** |

# Exploratory analyses

## Rationale of the statistical analyses

As mentioned in the main text, we aimed at testing *unimodal* and *cross-modal* adjustments on 7-to-20-month-old infants depending on two predictions: (i) if infants show *unimodal* adjustment, they will produce fewer silent-visual signals when the mother is visually inattentive than when she is attentive and (ii) if infants show *cross-modal* adjustment, they will produce more audible-or-contact than silent-visual signals when the mother is inattentive compared to when she is attentive.

To reach that goal, we first had to eliminate or control for possible confound variables related to the naturalistic conditions of observation. We therefore run two types of preliminary analyses; (1) stage 1 aimed at controlling the “nuisance” of confound variables and (2) stage 2 aimed at testing the effect of various aspects of maternal attention operationalized as visual attention (attentive/inattentive), infant-directed speech (talking/silent) and physical contact (yes/no).

In these two cases, we relied on Generalized Linear Mixed Models on proportion data (hereafter: GLMM; Bolker et al., 2009; Harrison, 2015). GLMMs were conducted with a binomial error distribution and the *logit link* function to determine which factors most affected the use of the different signal modalities, i.e., use of audible, silent-visual or contact signals. The dependent variable was the proportion of signals in a given modality for each infant corresponding to the *number of signals of one modality* /*total number of signals produced* (i.e., sum of tactile, audible and silent-visual signals).

## Nuisance control of confound variables

To account for variation in the outcome variable caused by “nuisance” independent variables that were not of interest in this study, we sought to identify which random structure would better control for these undesirable sources of variation (e.g., the typical example is the identity of an individual that can cause undesirable variation and adding it as a random effect allows to control for the pseudoreplication of using the same individual) (Theobald, 2018). To this end, many independent variables were integrated as potential random effects. We tested models with these following random effects, one by one, to identify the best fitting random structure for each dependant variable:

- *The sex* of the infant (male/female),
- The type *of daily care* of the infant (nursery/ nanny/housewife mother),
- Whether *breastfeeding* had occurred or not before (yes/no),
- The number of *siblings* (0/1/2),
- The *family configuration* referring to a nuclear family or not (yes/no),
- The *order of the observation* (first or second observation),
- The *time of meal* referring to the time of the observation (before, during or after meal),
- The *distance to the mother* referring to the distance between the mother and the infant (*close*: the mother is less than an arm's length from her infant; *far*: the mother is more than an arm's length from her infant; *out-of-sight*: the mother is totally out of sight of her infant).

The best fitting random structure was identified for each dependant variable thanks to the lowest AIC and was used in further analyses. Furthermore, the models showing convergence issues were excluded from the model selection.

Results of model fitting

For silent-visual, audible and tactile signals, random structure included random slopes of *time of meal* and random intercepts for the identity of the infant signaller (*silent-visual signals*: AICc = 2520.25; Chi-square test of the log-likelihood ratio with null model, χ2 = 205.95, df = 5, p < 0.001; *wi* = 1, *audible signals*: AICc = 2694.41; Chi-square test of the log-likelihood ratio with null model, χ2 = 205.47, df = 5, p < 0.001; *wi* = 1, *tactile signals*: AICc = 747.75; Chi-square test of the log-likelihood ratio with null model, χ2 = 70.632, df = 5, p < 0.001; *wi* = 1).

## Tests of maternal attention variables (Table S6)

GLMM analysis aimed at identifying which maternal attention cues, among maternal visual attention, IDS and physical contact, influenced the proportion of use of each signal modality most.

The dependent variables were the proportion of signals in a given modality for each infant (i.e., tactile, audible or silent-visual) corresponding for example to the *number of silent-visual signals* /*total number of signals produced* (i.e., sum of tactile, audible and silent-visual signals). Fixed effects were the factors of interest (i.e., maternal visual attention, IDS, physical contact and infant’s age in months). Random structures were the ones described above. The models were implemented using the *glmmTMB* function of the R package *glmmTMB* version 1.1‐23 with the optimizer function of *aictab* of the R package *AICcmodavg* to calculate AICc, which is a transformation of the Akaike Information Criterion (AIC) used for a small sample. For the model selection, the models with the lowest AIC values are considered the best-fitting models and are compared to a null model with an anova test. Also, we considered models within a ΔAIC of 2 are considered to be equally good (Burnham & Anderson, 2004).

Results of model fitting

The proportion of silent-visual signals was affected by maternal visual attention, IDS and mean age as evidenced by the unique best fitting model (AICc =2324.81; Chi-square test of the log-likelihood ratio with null model, χ2 = 205.64, df = 5, p < 0.001; wi = 0.99; see Table S6). The proportion of audible signals was also affected by maternal visual attention, IDS and mean age as evidenced by the unique best fitting model (AICc =2488.86; Chi-square test of the log-likelihood ratio with null model, χ2 = 215.73, df =5, p < 0.001; *wi* = 0.86; see Table S6). The proportion of tactile gestures was affected by maternal physical contact, visual attention and IDS as evidenced by the best fitting models. Indeed, one main factor model and two interactive models fitted equally well; infants showed differential tactile signaling according to maternal physical contact (AICc =731.46; Chi-square test of the log-likelihood ratio with null model, χ2 = 18.495, df = 1, p < 0.001; *wi* = 0.41; see Table S5), and the same result was found with the additional effect of maternal visual attention (AICc =731.94; Chi-square test of the log-likelihood ratio with null model, χ2 = 22.119, df = 3, p < 0.001; *wi* = 0.32; see Table S5) or IDS (AICc =733.34; Chi-square test of the log-likelihood ratio with null model, χ2 = 20.72, df = 3, p < 0.001; *wi* = 0.16; see Table S5). Regardless of the sensory modality of the infant’s signal, maternal visual attention, infant’s age and IDS were the most likely determining factors. These factors were therefore kept for further analysis based on predictions related to *unimodal* and *cross-modal* adjustment that were tested with non-parametric statistics (see the main text). All the non-parametric tests detailed in the main text were conducted twice, on data (i) with maternal IDS (‘talking mother’), and (ii) without it (‘silent mother’).

**Table S6. Summary of the models fitted with the proportion of each signal modality as dependent variable (n=8367).** Interactions between two effects are represented by colons; bold characters indicate the best fitting models based on the lowest AICc; k is the number of parameters (Burnham & Anderson, 2004; Symonds & Moussalli, 2011) in the model; ΔAICc gives the difference in AICc between each model and the best fitting model; AICcWt is Akaike weight corresponding to the probability that a given model is the best approximating model.

|  |  | **Silent-visual signals**  (random effect^(1)^: Time of meal\|ID) | | | | **Audible signals**  (random effect^(1)^: Time of meal\|ID) | | | | **Tactile signals**  (random effect^(1)^: Time of meal\|ID) | | | |
| --- | --- | --- | --- | --- | --- | --- | --- | --- | --- | --- | --- | --- | --- |
| **Model type** | **fixed effects** | **K** | **AICc** | **ΔAICc** | **AICcWt** | **K** | **AICc** | **ΔAICc** | **AICcWt** | **K** | **AICc** | **ΔAICc** | **AICcWt** |
| Null | Null | 7 | 2520.25 | 195.45 | 0.00 | 7 | 2694.41 | 205.54 | 0.00 | 7 | 747.91 | 16.45 | 0.00 |
| Single effect | Visual attention (VisualMot) | 8 | 2439.30 | 114.49 | 0.00 | 8 | 2603.67 | 114.81 | 0.00 | 8 | 749.89 | 18.43 | 0.00 |
|  | Physical contact (TactileMot) | 8 | 2512.54 | 187.74 | 0.00 | 8 | 2660.02 | 171.16 | 0.00 | 8 | 731.46 | 0.00 | 0.41^(2)^ |
|  | IDS | 8 | 2378.77 | 53.97 | 0.00 | 8 | 2544.48 | 55.62 | 0.00 | 8 | 749.19 | 17.73 | 0.00 |
|  | Age mean (in months) | 8 | 2522.28 | 197.47 | 0.00 | 8 | 2696.31 | 207.45 | 0.00 | 8 | 749.80 | 18.34 | 0.00 |
| Interactive | VisualMot:Age mean | 10 | 2425.42 | 100.61 | 0.00 | 10 | 2596.01 | 107.15 | 0.00 | 10 | 751.34 | 19.88 | 0.00 |
| effect | TactileMot:Age mean | 10 | 2515.82 | 191.02 | 0.00 | 10 | 2663.67 | 174.80 | 0.00 | 10 | 735.11 | 3.65 | 0.07 |
|  | IDS:Age mean | 10 | 2381.29 | 56.49 | 0.00 | 10 | 2547.26 | 58.40 | 0.00 | 10 | 751.38 | 19.92 | 0.00 |
|  | VisualMot:TactileMot | 10 | 2431.97 | 107.16 | 0.00 | 10 | 2574.74 | 85.88 | 0.00 | 10 | 731.94 | 0.48 | 0.32^(2)^ |
|  | VisualMot:IDS | 10 | 2335.28 | 10.48 | 0.01 | 10 | 2492.45 | 3.58 | 0.14 | 10 | 753.09 | 21.63 | 0.00 |
|  | IDS:TactileMot | 10 | 2378.47 | 53.66 | 0.00 | 10 | 2522.19 | 33.33 | 0.00 | 10 | 733.34 | 1.88 | 0.16^(2)^ |
|  | VisualMot,TactileMot:Age mean | 12 | 2423.23 | 98.42 | 0.00 | 12 | 2571.67 | 82.80 | 0.00 | 12 | 736.51 | 5.05 | 0.03 |
|  | VisualMot,IDS: Age mean | 12 | 2324.81 | 0.00 | 0.99^(2)^ | 12 | 2488.86 | 0.00 | 0.86^(2)^ | 12 | 753.26 | 21.80 | 0.00 |
|  | IDS,TactileMot: Age mean | 12 | 2382.67 | 57.87 | 0.00 | 12 | 2533.34 | 44.47 | 0.00 | 12 | 737.70 | 6.23 | 0.02 |

^(1 )^ *ID* refers to the individual infant and *Time of meal* refers to the time of the observation (before, during or after meal).

^(2)^ Comparison and significance with the null model (***: p-value<0.001).

# Additional illustration (Figure S1)


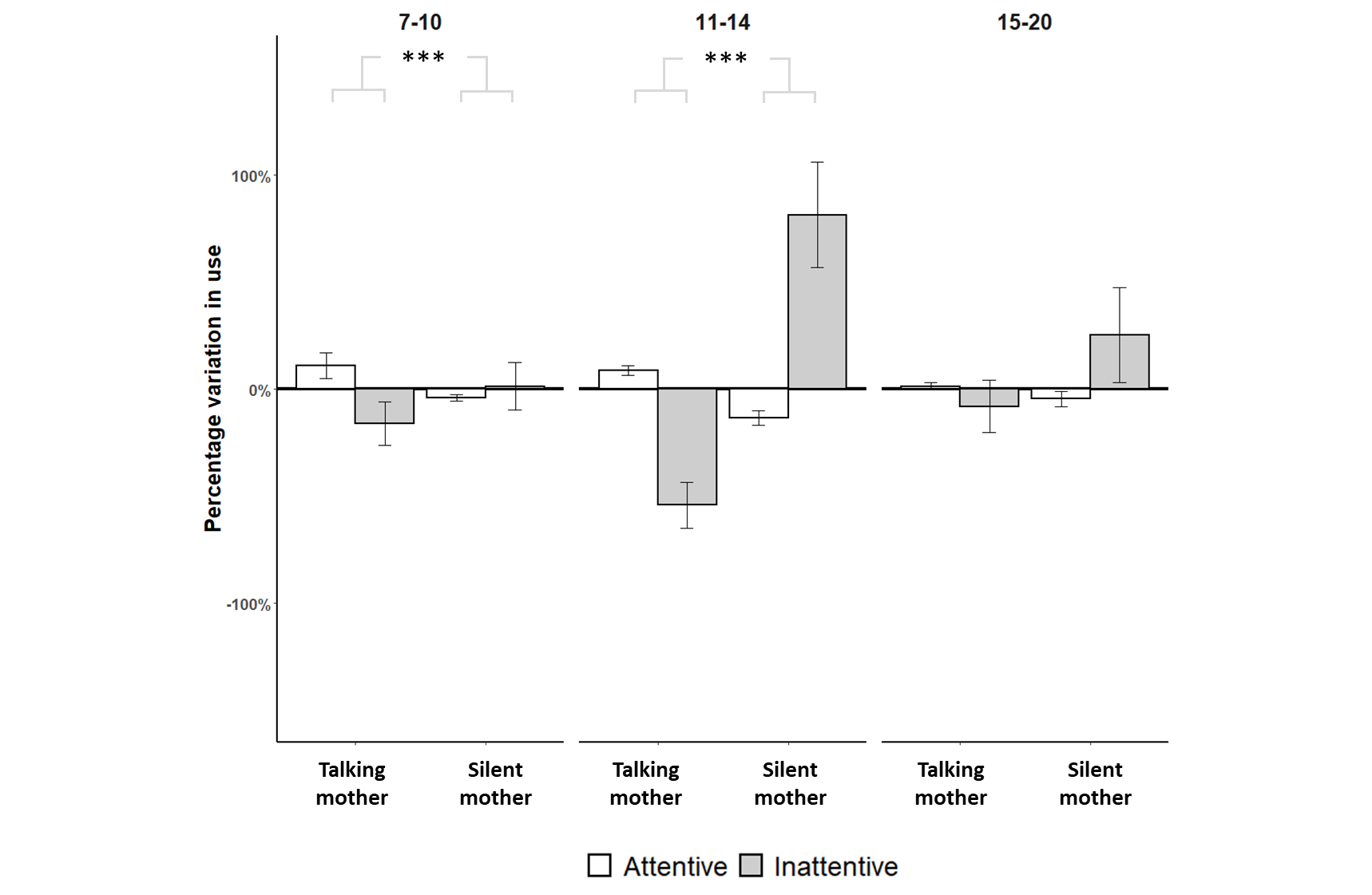


**Figure S1. Variation in use of silent‐visual gestures with respect to IDS by age range and maternal visual attention** (N = 11, *p < 0.05. ***p < 0.001). The deviations above and below the zero‐line show changes (plus standard error bar) in the distribution of silent‐visual gestures across maternal visual condition and IDS, from the overall average use of silent‐visual gestures in this condition regardless of IDS.

*Note: To represent the IDS effect on the adjustment of the silent-visual gestures towards the maternal visual attention state, we calculated the percentage deviation in the variation in the use of silent-visual gestures in attentive condition as compared with the use of silent-visual gestures in inattentive condition for each IDS condition and age range. The deviation was calculated for each individual by (β/α − 1) × 100 with β= number of silent-visual gestures in attentive and IDS condition /total number of silent-visual gestures used in the condition IDS and α = number of silent-visual gestures in attentive condition /total silent-visual gestures in the sample. Then, we calculated the mean by age group.*
